# Expressing a cytosolic pyruvate dehydrogenase complex to increase free fatty acid production in *Saccharomyces cerevisiae*

**DOI:** 10.1186/s12934-020-01493-z

**Published:** 2020-12-10

**Authors:** Yiming Zhang, Mo Su, Ning Qin, Jens Nielsen, Zihe Liu

**Affiliations:** 1 Beijing Advanced Innovation Center for Soft Matter Science and Engineering, College of Life Science and Technology, Beijing University of Chemical Technology, No.15 North Third Ring Road East, Chaoyang District, Beijing, 100029 People’s Republic of China; 2 Department of Biology and Biological Engineering, Chalmers University of Technology, Gothenburg, Sweden; 3 BioInnovation Institute, Ole Maaløes Vej 3, 2200 Copenhagen N, Denmark

**Keywords:** The pyruvate dehydrogenase complex, Free fatty acids, Redox factor, *Saccharomyces cerevisiae*

## Abstract

**Background:**

*Saccharomyces cerevisiae* is being exploited as a cell factory to produce fatty acids and their derivatives as biofuels. Previous studies found that both precursor supply and fatty acid metabolism deregulation are essential for enhanced fatty acid synthesis. A bacterial pyruvate dehydrogenase (PDH) complex expressed in the yeast cytosol was reported to enable production of cytosolic acetyl-CoA with lower energy cost and no toxic intermediate.

**Results:**

Overexpression of the PDH complex significantly increased cell growth, ethanol consumption and reduced glycerol accumulation. Furthermore, to optimize the redox imbalance in production of fatty acids from glucose, two endogenous NAD^+^-dependent glycerol-3-phosphate dehydrogenases were deleted, and a heterologous NADP^+^-dependent glyceraldehyde-3-phosphate dehydrogenase was introduced. The best fatty acid producing strain PDH7 with engineering of precursor and co-factor metabolism could produce 840.5 mg/L free fatty acids (FFAs) in shake flask, which was 83.2% higher than the control strain YJZ08. Profile analysis of free fatty acid suggested the cytosolic PDH complex mainly resulted in the increases of unsaturated fatty acids (C16:1 and C18:1).

**Conclusions:**

We demonstrated that cytosolic PDH pathway enabled more efficient acetyl-CoA provision with the lower ATP cost, and improved FFA production. Together with engineering of the redox factor rebalance, the cytosolic PDH pathway could achieve high level of FFA production at similar levels of other best acetyl-CoA producing pathways.

## Background

Bioproduction of fatty acids and their derivatives serves as a sustainable route to alleviate the dilemma between the growing demands of high-energy–density fuels and concerns on their environmental impacts. However, fatty acids are involved in several essential cellular processes, such as membrane synthesis, energy storage, and protein modification, and their levels are therefore tightly regulated to ensure in vivo homeostasis [1]. Intensive efforts have been carried out to improve the titer, rate and yield (TRY) of fatty acid-derived biofuels, focusing mainly on the deregulation of fatty acid metabolism and redirection of carbon flux [2–4].

Regarding deregulation of fatty acid metabolism, deletions of dominant acyl-CoA synthetases (*FAA1*, *FAA4*), and fatty acyl-CoA oxidase (*POX1*) catalyzing the first step in β-oxidation, could significantly accumulate free fatty acids (FFAs) [5–7]. Moreover, deletions of main sterol ester formation genes (*ARE1*, *ARE2*), and phosphatidate phosphatase genes (*PAH1*, *DPP1*, *LPP1*) could also deregulate fatty acid synthesis and increase the production of total fatty acids [8]. Combining deletion of several lipid regulation mechanisms resulted in a reduced complexity of the fatty acid metabolic network, leading to enhanced FFA accumulation [8, 9].

Besides engineering lipid metabolic network, engineering of precursor supply and redox cofactor regeneration was also essential for carbon flux redirection towards FFA production. Each elongation cycle in fatty acid synthesis extends the backbone with a C2 unit and requires 2 molecules of NADPH as cofactor. Endogenous synthesis of cytosolic acetyl-CoA, the main C2 metabolite, is often limited and several heterologous enzymes have therefore been expressed to increase acetyl-CoA supply for FFA synthesis, including NAD^+^-dependent alcohol dehydrogenase (AdhE), NAD^+^-dependent acylating acetaldehyde dehydrogenase (EutE) [10], ATP citrate lyases (ACLs) [6, 7, 11–13], xylulose-5-phosphate phosphoketolase (XpkA) and phosphotransacetylase (Pta) [14]. Compared to these strategies, expression of cytosolic pyruvate dehydrogenase (PDH) is attractive because of its lower energy cost [15]. The PDH complex consists of three catalytic subunits, including pyruvate dehydrogenase (E1), dihydrolipoyl transacetylase (E2), and dihydrolipoyl dehydrogenase (E3), and requires four kinds of cofactors TPP, lipoate, FAD and NAD^+^. Among three PDH complexes that have been expressed in the cytosol of yeast, PDH from *Enterococcus faecalis* was demonstrated to enable complete replacement of the native pathway for cytosolic acetyl-CoA synthesis, and was less sensitive to high NADH/NAD^+^ ratios compared with other PDH complexes [16–18]. However, one concern with PDH expression for FFA production is the redox imbalance between redox couples of NAD^+^/NADH and NADP^+^/NADPH.

**Fig. 1:**
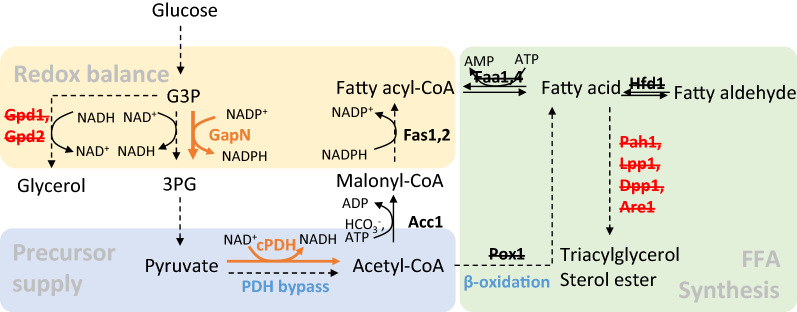
Schematic representation of engineering strategies for FFA production in *S. cerevisiae*.* G3P* glyceraldehyde-3-phosphate,* 3PG* 3-phospho-glycerate,* cPDH* cytosolic pyruvate dehydrogenase. A cytosolic PDH complex was evaluated in a *faa1Δ faa4Δ pox1Δ hfd1Δ* mutant YJZ08 for free fatty acid production, in combination with fatty acid metabolism engineering via *pah1Δ lpp1Δ dpp1Δ are1Δ* and redox cofactor engineering via *GPD* deletion and *GapN* expression

Previous studies showed that high glycerol production was often observed in FFA production strains, indicating a high NADH pressure [7]. Glycerol synthesis from dihydroxyacetone phosphate consists of two steps, catalyzed by NAD^+^-dependent glycerol-3-phosphate dehydrogenase (encoded by *GPD1* and *GPD2*) and glycerol-3-phosphate phosphatase (encoded by *GPP1* and *GPP2*), respectively (Fig. 1). Glycerol accumulation is also an issue in bioethanol production and has therefore been studied for years. Several studies found that glycerol formation could be decreased with altered redox-cofactor specificity [11, 19–22], and could be eliminated through inactivation of both *GPD1* and *GPD2* with the expense of a dramatic reduction in cell growth [23]. As alternatives, an NADP^+^-dependent glyceraldehyde-3-phosphate dehydrogenase (GapN) was demonstrated to enable rescuing the negative effects and improve ethanol yield [24, 25]. Moreover, since GapN irreversibly converts glyceraldehyde-3-phosphate to 3-phosphoglycerate and produces NADPH (Fig. 1), it was reported to increase the synthesis of NADPH demanding bioproducts, including 3-hydroxypropionic acid (3-HP) and polyhydroxybutyrate (PHB) [26, 27]. Therefore, it could potentially eliminate glycerol accumulation and increase FFA production by interruption of glycerol synthesis and expression of *GapN*.

The goal of this study is to investigate the effect of expressing the cytosolic PDH complex on FFA production in yeast strains with deregulated fatty acid metabolism and optimized cytosolic redox balance (Fig. 1). The strategies reported here may shed lights for production of other acetyl-CoA and NADPH demanding chemicals as well.

## Results and discussion

### Expression of a bacterial PDH complex to increase the acetyl-CoA supply

**Fig. 2:**
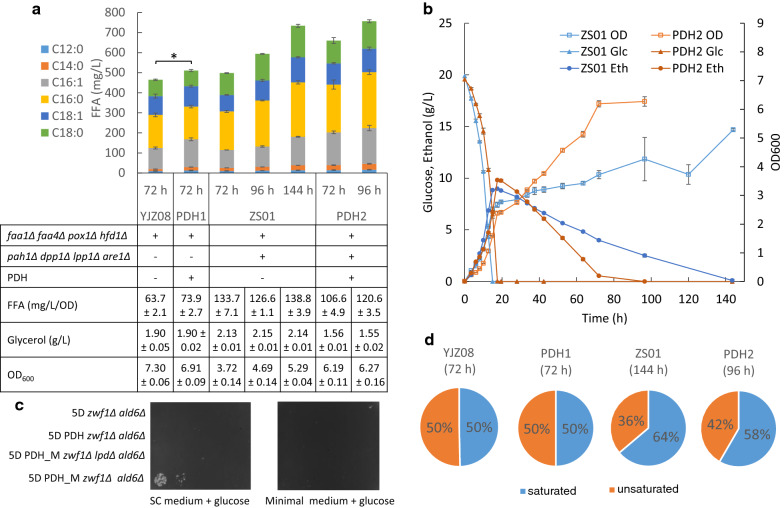
The expression of PDH improved FFA production in yeast with simplified lipid networks. **a** FFA production with expression of PDH complex in YJZ08. **b** Growth profile with expression of PDH complex in ZS01. **c** Growth assay of engineered strains with and without cytoplastic PDH in glucose medium. **d** Distribution of saturated and unsaturated FFAs (C16 and C18) in engineered strains. Statistical analysis was performed using one-tailed Student’s *t*-test (*p < 0.05). All data represent the mean ± SD of biological triplicates

A FFA producing strain YJZ08 with four gene deletions (*faa1Δ faa4Δ pox1Δ hfd1Δ*) was used as the background strain for cytosolic PDH evaluation [6]. The three subunits of PDH complex, encoded by *pdhA*, *pdhB*, *aceF* and *lpd*, and lipoate ligases from *E. faecalis*, encoded by *lplA* and *lplA2* [16], were integrated into the two genome sites Chr XII-5 and Chr XI-3 [28] of, yielding strain PDH1. As shown in Fig. 2, YJZ08 with deregulated FA synthesis produced FFA at 458.9 mg/L in 72 h in minimal medium with 20 g/L glucose, which was consistent with previous studies [6, 9]. Meanwhile, the constructed strain PDH1 produced 512.7 mg/L FFA at 72 h (Fig. 2A) with a lower ethanol yield (Additional file: Table S1). From the growth data, we found that *E. faecalis* PDH reduced the maximum specific growth rate from 0.33 h^−1^ to 0.28 h^−1^ but didn’t result in much difference in the final biomass. PDH1 accumulated 1.90 g/L glycerol, compared with 1.90 g/L glycerol with YJZ08 (Fig. 2a), suggesting that cytosolic PDH did not cause an NADH burden in YJZ08.

Theoretically, a combination of the PDH route with the phosphoketolase (PK) pathway can result in a higher maximum FFA yield [15], since the PDH route could produce acetyl-CoA with low energy cost and the PK pathway could produce acetyl-CoA with low carbon loss. Moreover, the PK pathway utilizes xylulose-5-phosphate from the pentose phosphate (PP) pathway as the substrate, and it may thermodynamically help to pull the carbon flux towards the PP pathway and increase NADPH generation. However, we tested the integration of xylulose-5-phosphate phosphoketolase from *Leuconostoc mesenteroides* [29] and phosphotransacetylase from *Clostridium kluyveri* [30] into both YJZ08 and PDH1, and did not find significant improvement in cell growth and FFA synthesis (Additional file 1: Figure S1), probably because of the strict control of carbon flux through the PP pathway [31] or inefficient phosphoketolase enzymes [29].

Moreover, the yeast ZS01 strain with simplified lipid metabolic networks and redirected flux towards FFA synthesis was constructed by deletion of *PAH1*, *DPP1*, *LPP1* and *ARE1* in YJZ08. Then, the genes encoding the PDH complex, composing of *pdhA*, *pdhB*, *aceF*, *lpd*, *lplA* and *lplA2*, were integrated into the chromosome of ZS01, yielding strain PDH2. As shown in Fig. 2, when cultivated in minimal medium with 20 g/L glucose, ZS01 accumulated 497.9 mg/L FFAs at 72 h with a high productivity of 133.7 mg/L/OD, which was consistent with previous studies [8, 9]. After 72 h, ZS01 accumulated more FFAs since there was ethanol remaining and the FFA titer reached 734.2 mg/L at 144 h when ethanol was exhausted (Fig. 2a). Interestingly, PDH2 produced 660.2 mg/L FFAs at 72 h and 756.3 mg/L FFAs at 96 h when ethanol was completely exhausted. In PDH2, the faster FFA production rate accompanied with a faster ethanol consumption rate and lower glycerol level, indicating that cytosolic PDH may alleviate NADH burden and speed up cell growth and FFA production (Fig. 2b), probably due to the lower energy cost.

To address the possible mismatch between acetyl-CoA supply, NADH production and NADPH consumption for FFA synthesis, we evaluated whether PDH from *E. faecalis* might utilize NADP^+^ as well as NAD^+^ as redox cofactors in a *zwf1Δ ald6Δ* mutant, which hardly grew on glucose medium due to lack of ability to produce cytosolic NADPH [32]. The results showed that cytosolic PDH expression could not rescue cell growth of the *zwf1Δ ald6Δ* mutant on glucose (Fig. 2c). A previous study showed that cofactor specificity of *E. coli* PDH could be converted from NAD^+^ to NADP^+^ via seven amino acid mutations in E3 [33, 34]. The alignment result of E3 proteins from *E. faecalis* and *E. coli* suggested that the mutated amino acids are highly conserved, as shown in Additional file 1: Figure S2a. Expression of the mutated PDH recued the growth on glucose and disruption of E3 encoding gene *lpd* suspended the growth recovery, suggesting the mutated PDH might utilize NADP^+^ as redox cofactor. Growth assay on minimal glucose medium didn’t show similar results, which might be caused by its poor activity (Fig. 2c). However, these E3 mutations didn’t increase FFA titer, but reduced the titer to 439.5 mg/L, comparable with YJZ08 (Additional file 1: Figure S2b and Table S1). The FFA results indicated that the mutations resulted in enzymatic activity lost, probably due to unsuccessful protein assembly. To improve its enzymatic activity towards NADP^+^, rational design based on PDH structures or directed evolution will be required for further investigation. As *E. faecalis* PDH has been demonstrated to be a functional enzyme in *E. coli* and yeast at both aerobic and anaerobic conditions [16, 35], a novel PDH complex with NADP^+^ preference will clearly be valuable for bioproduction of acetyl-CoA derived chemicals.

It was also observed that, PDH expression altered the distribution of saturated and unsaturated FFAs (C16 and C18) in PDH2, but not in PDH1 (Fig. 2d). The double bonds in unsaturated FFAs were formed by Delta-9 fatty acid desaturase, encoded by *OLE1*, through an oxygen-dependent mechanism that requires reducing equivalents from NADH. Thus, the increase in unsaturated FFA ratio might also indicate that the functional PDH resulted in cytosolic redox changes without imposing an increased NADH burden, due to its insensitive feature to high NADH/NAD^+^ ratios [35–37].

### Balance of cytosolic redox factors for FFA production

NAD and NADP redox couples are crucial to maintain cellular redox hemostasis, with lower cytosolic NADH/NAD^+^ ratios and higher NADPH/NADP^+^ ratios due to their different functions [38]. In FFA production strains, over-production of NADH resulted in glycerol accumulation, while NADPH as the cofactor required for fatty acid elongation was limited. Thus, the balance between NAD and NADP redox couples would be important for FFA production.

**Fig. 3:**
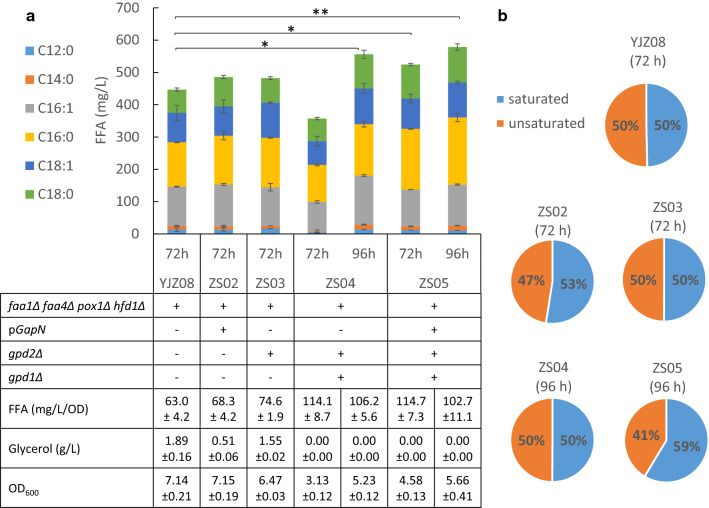
FFA production with *GapN* expression and *GPD* deletions. **a** FFA production obtained with engineered strains. **b** Distribution of saturated and unsaturated FFAs (C16 and C18) in the engineered strains. Statistical analysis was performed using one-tailed Student’s *t* test (*p < 0.05, **p < 0.01). All data represent the mean ± SD of biological triplicates

To balance the redox state of the cell and further improve FFA synthesis, NADP^+^-dependent glyceraldehyde-3-phosphate dehydrogenase (*GapN*) from *Streptococcus mutans* [39] was expressed on a multi-copy plasmid pGapN in the FFA production strain YJZ08, yielding strain ZS02. As shown in Fig. 3, *GapN* expression resulted in reduced glycerol accumulation and increased FFA production.

We further balanced cytosolic redox conditions through interruption of glycerol synthesis via glycerol-3-phosphate dehydrogenase (GPD) deletions. Since *GPD1* was regulated by osmotic stress [23], *GPD2* was first deleted in YJZ08, and the resulting strain ZS03 showed decreased glycerol accumulation, and increased FFA titer and productivity (Fig. 3a). When both *GPD1* and *GPD2* were deleted, the specific growth rate of the resulted strain ZS04 decreased dramatically, which was consistent with previous studies [23]. The final FFA titer of ZS03 was 556.0 mg/L, 23.6% higher than YZJ08. When *GapN* was introduced into ZS04, the impaired growth was improved, and FFA production at 72 h improved significantly. The final FFA titer of ZS05 reached 578.7 mg/L at 96 h, which was 28.6% higher than YJZ08 and the productivity was comparable with ZS04.

Similarly, the distributions of saturated and unsaturated FFA also varied in the constructed strains (Fig. 3b). *GPD* deletion did not alter the distributions, whereas *GapN* expression increased the ratios of saturated FFAs. With *GapN* expressed in YJZ08, the percentage of saturated FFAs increased slightly from 50 to 53%, with the increases in the titers of C16:0 and C18:0 but no increases of C16:1 and C18:1. When *GapN* expressed in *gpd1Δ gpd2Δ* mutant, in ZS05 the percentage of saturated FFAs further increased to 59%, indicating the possible redox changes between NAD and NADP redox couples.

### Increased provision of acetyl-CoA and NADPH improved FFA production

**Fig. 4:**
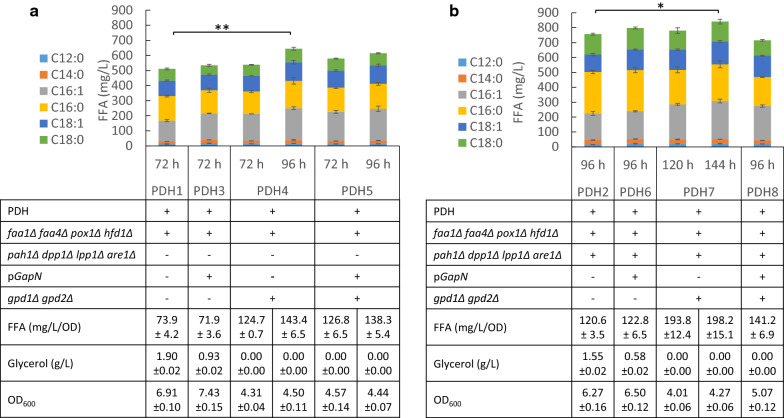
FFA production with cytosolic PDH and cofactor engineering. All data represent the mean ± SD of biological triplicates

Plasmid pGapN was transformed into PDH1 and PDH2, yielding PDH3 and PDH6, respectively. As shown in Fig. 4, the FFA production in PDH3 and PDH6 increased to 534.0 and 797.6 mg/L, respectively. Also, glycerol accumulation levels in PDH3 and PDH6 reduced to 0.93 and 0.58 g/L, respectively. The decrease in glycerol level and increase in FFA titer in both PDH3 and PDH6 were similar with those observed in ZS02.

Similarly, when *GPD1* and *GPD2* were deleted in PDH1 and PDH2, the resulting strains PDH4 and PDH7 exhibited decreased growth rates, but increased FFA production to 664.0 and 840.5 mg/L, respectively (Fig. 4). Finally, when *GapN* was introduced into PDH4 and PDH7, yielding PDH5 and PDH8, the impaired cell growth improved, whereas the FFA production decreased to 614.0 and 715.3 mg/L, respectively (Fig. 4). The improved cell growth and decreased FFA titer upon GapN expression indicated a possible competition for the supply of the precursors and redox factors. Previous studies with heterologous acetyl-CoA pathways suggested that the ACL pathway is promising for production of fatty acid and their derivatives [6, 7, 10, 11, 14]. ACL expression with enhanced fatty acid synthesis resulted in 81% higher FFAs than YJZ08 [6, 7], and in this study the titer of PDH7 was 83.2% higher compared to YJZ08, similar with ACL expression combined with other engineering efforts.

**Fig. 5:**
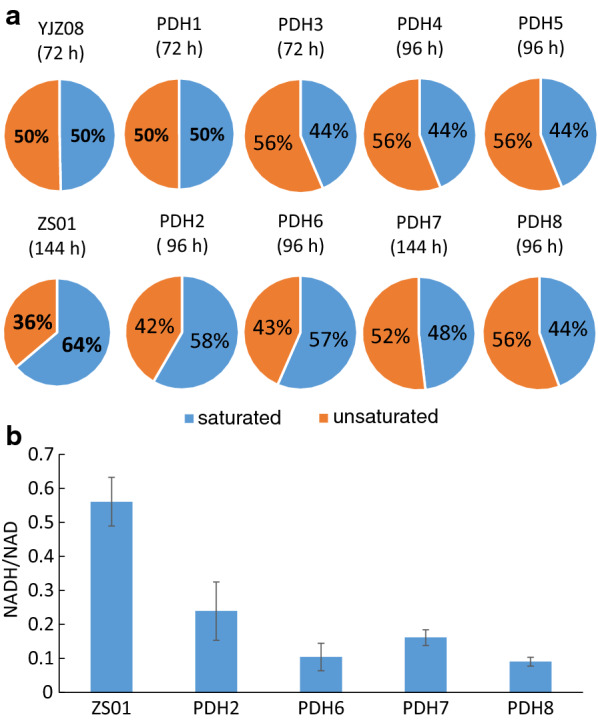
Distribution of saturated and unsaturated FFAs (C16 and C18) in engineered strains. All data represent the mean ± SD of biological triplicates

Regarding the distribution of saturated and unsaturated FFAs (C16 and C18) of engineered PDH strains, it was found that PDH expression significantly increased the ratios of unsaturated FFAs (Fig. 5a), even with efforts for redox cofactor rebalance. These changes might be because of the PDH complex with excess NADH produced and its consistent activity under high NADH/NAD^+^ ratios. The PDH complex may be a potential target to increase the unsaturation of the FFAs and their derivatives, besides manipulations on Delta-9 fatty acid desaturase Ole1 reported to control the fatty acid saturation in previous studies [40, 41].

The intracellular NADH/NAD^+^ ratios of the engineered PDH strains were lower than that of ZS01 (Fig. 5b), suggesting the PDH complex alleviated NADH burden, as we conferred from cell growth and FFA production. *GapN* expression could further relieve the NADH burden, as the NADH/NAD^+^ ratio of PDH6 and PDH8 was lower than that of PDH2, and the ratio of PDH8 lower than PDH7.

## Conclusions

The expression of the PDH complex from *E. faecalis* in the yeast cytosol enabled efficient acetyl-CoA provision with low ATP cost, and improved FFA production in the titer, productivity and rate. Rebalancing NAD and NADP redox couples further improved FFA production in PDH expressing strains. Briefly, the *GapN* expression seemed to increase FFA production via the growth improvement, and *GPD* deletion resulted in dramatically decreased cell growth probably due to the high cytosolic NADH burden. The strain PDH7 with PDH expression and *GPD* deletion could achieve a similarly high level of FFA production as other best acetyl-CoA producing pathways, and could potentially engineered for better performances by enhancing fatty acid synthesis and fine-tuning carbon metabolism. Moreover, the increase in FFA unsaturation in PDH strains suggested that the PDH complex can be further engineered for the production of unsaturated fatty acids and their derivatives.

## Materials and methods

### Construction of plasmids and strains

The plasmids, strains, and primers used and constructed in this study are listed in Additional file 2: Dataset S1. All yeast strains were constructed using GTR-CRISPR system as described in [8]. For gene integration and deletion, donor DNA containing 50 bp of homology arms and Cas9 plasmid containing gRNA targeting sequence were co-transformed using the electroporation method as described in [42].

Six genes for PDH pathway from *Enterococcus faecalis* [16] were synthesized by Sangon Company (Shanghai, China) and then cloned in pUC57 vectors, yielding plasmids pPdhB, pPdhA, pLpd, pAceF, pLplA and pLplA2, repectively. *lplA* and *lplA2* were cloned using Golden Gate into the vector pAMPori with primers PP1-5, yielding pLplA_A2. These six genes were integrated into two sites XI-3 and XII-5 as described in [28]. *lplA* and *lplA2* were integrated at XI-3 using the Cas9 plasmid pCas9_XI-3.3 and donor DNA amplified from pLplA_A2 with primers PP6 and PP7. *pdhB*, *pdhA*, *lpd* and *aceF* were integrated at the XII-5 site simultaneously using pCas9_XII-5 and donor DNA amplified from the corresponding plasmids with primers PP8-15.

Codon-optimized *GapN* from *Streptococcus mutans* [39] was synthesized by Sangon Company (Shanghai, China) and cloned in pAMPori vector using Golden Gate assembly with primers PP16-23, yield pGapN. *GapN* was then integrated into XI-1 using pCas9_XI-1 and donor DNA amplified with primers PP24 and PP25 from pGapN.

*GPD2* was deleted using pCas9_gpd2 and donor DNA amplified with primers PP26 and PP27. *GPD1* was deleted using pCas9_gpd1 and donor DNA amplified with primers PP28 and PP29. *PAH1*, *DPP1*, *LPP1* and *ARE1* were simultaneously deleted using pCas9_PDLA and donor DNA fragments with primers PP30-PP37. *ZWF1* was deleted using pCas9_zwf1 and donor DNA amplified with primers PP38 and PP39. *ALD6* was deleted using pCas9_ald6 and donor DNA amplified with primers PP40 and PP41. *LPD* was deleted using pCas9_lpd and donor DNA amplified with primer PP42 and PP43.

All Cas9 plasmids containing gRNAs for target genes and were constructed using Golden Gate assembly. Take pCas9_gpd2 as an example, PCR fragments amplified from pScURA with primer PP47, containing 20 bp gRNA targeting sequence, were assembled into pCas plasmid [8].

### Media and cultivation conditions

*E. coli* strains were cultured at 37 °C in LB medium, composed of 5 g/L yeast extract,10 g/L peptone, and 10 g/L NaCl, and 80 mg/mL ampicillin was added when needed.

Yeast strains were cultured either in YPD medium composed of 10 g/L yeast extract, 20 g/L peptone and 20 g/L glucose, or SC-URA medium for the selection of strains containing URA3-based plasmids, composed of 5 g/L (NH_4_)_2_SO_4_, 1.7 g/L yeast nitrogen base without amino acids, 1.914 g/L amino acid mixture without uracil, and 20 g/L glucose. 5-FOA plates were used to lose URA3-based plasmid, composed of 1.7 g/L yeast nitrogen base without amino acids, 5 g/L (NH_4_)_2_SO_4_, 1.914 g/L amino acid mixture without uracil, 0.05 g/L uracil, 1 g/L 5-FOA, 25 g/L agar. All solid plates contained 15 g/L agar except for 5-FOA plates.

Minimal medium was used for cultivation in shake flasks for FFA production, composed of 5 g/L (NH_4_)_2_SO_4_, 14.4 g/L KH_2_PO_4_, 0.5 g/L MgSO_4_·7H_2_O, trace metal solution and vitamin solution and 20 g/L glucose, and 40 mg/L histidine or 60 mg/L uracil was added when needed. For all PDH strains, minimal medium was supplemented with 100 mg/mL lipoic acid.

Yeast strains were cultured at 30 °C. For shake flask cultivation, yeast cells were precultured in falcon tubes with 5 mL minimal medium for 24 h, and then inoculated into 100 mL shake flasks with 30 mL medium with initial OD_600_ of 0.1.

### Measurement of biomass and extracellular metabolites

Biomass was measured by optical density at 600 nm (OD_600_) with a GENESYS 30 Visible Spectrophotometer (Thermo Electron Scientific, Madison, USA).

Extracellular metabolites including glucose, ethanol and glycerol were measured by HPLC (Shimadzu LC-20AT, Japan) equipped with RID and UV detectors at 210 nm, using 5 mM H_2_SO_4_ as eluent and Aminex HPX-87H column (Bio-Rad) at 65 °C with a flow rate of 0.6 mL/min. The injection volume was 10 μL. A mixture composed of 20 g/L glucose, 15 g/L ethanol, 0.5 g/L pyruvate, 0.5 g/L succinate, 2 g/L glycerol 2 g/L acetate was diluted into 6 gradients with eluent and used as HPLC standards.

### Extraction, measurement and analysis of FFAs

Total FFAs including extracellular and intracellular FFAs were extracted and measured as described in [6]. Specifically, 200 μL of cell culture or diluted cell culture was transferred into a glass vial, and 10 μL 40% tetrabutylammonium hydroxide and 200 μL methylation reagent was added immediately. Methylation reagent contained 200 mM methyl iodide as methyl donor and 12.5 mg/L pentadecanoic acid as an internal standard. The mixture was shaken for 30 min, and then centrifuged at 5000 g for 3 min. 100 μL of dichloromethane layer with extracted methyl esters was transferred into a GC vial, evaporated 3 h, and resuspended in 100 μL hexane. Then samples were analyzed by GC–MS (QP2020, Shimadzu, Japan) with a DB-5MS column (30 m × 0.250 mm × 0.25 μm, Agilent). The program was set as follows: 40 °C 2 min; ramp to 130 °C at 5 °C/min; raise to 280 °C at 10 °C/min; 280 °C 3 min. The temperatures of the inlet, mass transfer line and ion source were set at 280, 300, 230 °C, respectively. Flow rate of carrier gas was 3.0 mL/min and the injection volume was 1 μL. The data were collected in the full inspection mode (50–650 m/z), analyzed using GCMS solution 4.4 software and quantified with a standard curve. The standard curve was calibrated using methyl ester standards (F.A.M.E mix, Sigma) for even-chain fatty acids (C8-C22) and pentadecanoic acid (C15) with 6 diluted gradients.

Samples for FFA analysis were harvested at different time points due to varied strain growth. The highest FFA titers were achieved when ethanol was completely consumed. Statistical analysis was performed using one-tailed Student’s t test using Microsoft Excel.

### Quantification of intracellular NADH/NAD^+^

Cells were grown to mid-log phase and harvested by centrifugation at 11000 *g* for 1 min. Cell pellets were suspended with 400 μL of extraction buffer from the NADH/NAD^+^ Quantitation Kit (Sigma-Aldrich) and transferred to a new tube with 50 μL of acid-washed glass beads, and kept on dry ice for 10 min. Cells were then lysed with a Fastprep homogenizer and immediately put on ice for 2 − 3 min, and centrifuged at 17 000 g for 10 min. The supernatant was filtered using a Pierce protein concentrator (10 K MWCO PES, Thermo Scientific) to remove proteins according to the kit instruction. All steps between cell harvest and enzymatic assay were carried out at 0 °C. NADH and NAD^+^ were measured following the manufacturer’s instruction with a micro-plate reader at 450 nm.

## Supplementary Information

**Additional file 1:** **Table S1.** Ethanol production in engineered strains. **Figure S1.** FFA production with phosphoketolase pathway expressed in YJZ08 and PDH1. **Figure S2.** Mutations in E3 resulted in decreased FFA production.

**Additional file 2. Dataset S1. **Primers, strains and plasmids used and constructed in this study.

## Data Availability

All datasets generated and analyzed during this study are included in this published article and its supplementary information files.

## Abbreviations

PDH: Pyruvate dehydrogenase
FFA: Free fatty acid
GPD: NAD^+^-dependent glycerol-3-phosphate dehydrogenase
