# Supplementary material for: Expressing a cytosolic pyruvate dehydrogenase complex to increase free fatty acid production in Saccharomyces cerevisiae
Source: Microb Cell Fact. 2020 Dec 10;19:226. doi: 10.1186/s12934-020-01493-z (PMC7730738; doi:10.1186/s12934-020-01493-z)
Supplement: Supplementary file 1 — Additional file 1: Table S1. Ethanol production in engineered strains. Figure S1. FFA production with phosphoketolase pathway expressed in YJZ08 and PDH1. Figure S2. Mutations in E3 resulted in decreased FFA production. [file 12934_2020_1493_MOESM1_ESM.docx]

**Supplementary**

**Table S1.** Ethanol production in engineered strains. All data represent the mean ± SD of biological triplicates.

|  | YJZ08 | PDH1 | ZS01 | PDH2 |
| --- | --- | --- | --- | --- |
| Ethanol yield (g/g glucose) | 0.35±0.07 | 0.27±0.03 | 0.46±0.12 | 0.46±0.09 |


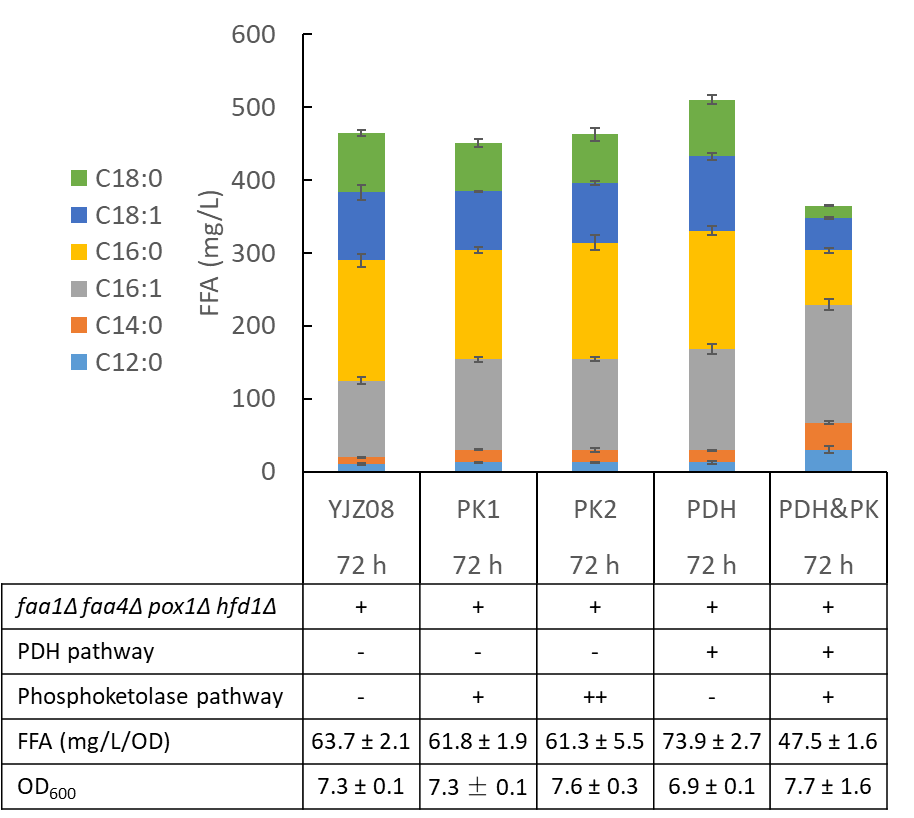


**Figure S1**. FFA production with phosphoketolase pathway expressed in YJZ08 and PDH1. Phosphoketolase enzymes xylulose-5-phosphate phosphoketolase and phosphotransacetylase were integrated into YJZ08 with a single copy or two copies, resulting in the strains PK1 and PK2, respectively. Phosphoketolase pathway was integrated into PDH1, resulting in the strain PDH&PK.


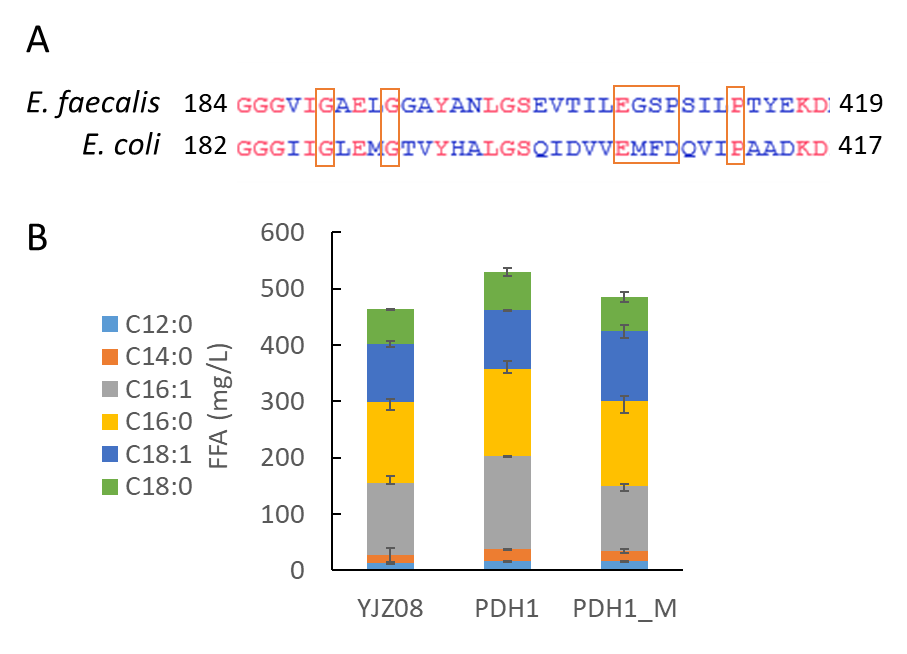


**Figure S2**. Mutations in E3 resulted in decreased FFA production. A) Alignment results of E3 proteins from *E. faecalis* and *E. coli*. Mutated amino acids were marked in red boxes. B) FFA production with mutated PDH complex expressed in YJZ08.
